# ALS mutant SOD1 interacts with G3BP1 and affects stress granule dynamics

**DOI:** 10.1007/s00401-016-1601-x

**Published:** 2016-08-01

**Authors:** Jozsef Gal, Lisha Kuang, Kelly R. Barnett, Brian Z. Zhu, Susannah C. Shissler, Konstantin V. Korotkov, Lawrence J. Hayward, Edward J. Kasarskis, Haining Zhu

**Affiliations:** 1 Department of Molecular and Cellular Biochemistry, University of Kentucky, Lexington, KY 40536 USA; 2 Math Science Technology Center, P L Dunbar High School, Lexington, KY 40513 USA; 3 Department of Neurology, University of Massachusetts Medical School, Worcester, MA 01655 USA; 4 Department of Neurology, University of Kentucky, Lexington, KY 40536 USA; 5 Lexington VA Medical Center, Research and Development, Lexington, KY 40502 USA

**Keywords:** ALS, SOD1, G3BP1, Stress granules, Neurodegeneration

## Abstract

Amyotrophic lateral sclerosis (ALS) is a fatal neurodegenerative disease. Mutations in Cu/Zn superoxide dismutase (SOD1) are responsible for approximately 20 % of the familial ALS cases. ALS-causing SOD1 mutants display a gain-of-toxicity phenotype, but the nature of this toxicity is still not fully understood. The Ras GTPase-activating protein-binding protein G3BP1 plays a critical role in stress granule dynamics. Alterations in the dynamics of stress granules have been reported in several other forms of ALS unrelated to SOD1. To our surprise, the mutant G93A SOD1 transgenic mice exhibited pathological cytoplasmic inclusions that co-localized with G3BP1-positive granules in spinal cord motor neurons. The co-localization was also observed in fibroblast cells derived from familial ALS patient carrying SOD1 mutation L144F. Mutant SOD1, unlike wild-type SOD1, interacted with G3BP1 in an RNA-independent manner. Moreover, the interaction is specific for G3BP1 since mutant SOD1 showed little interaction with four other RNA-binding proteins implicated in ALS. The RNA-binding RRM domain of G3BP1 and two particular phenylalanine residues (F380 and F382) are critical for this interaction. Mutant SOD1 delayed the formation of G3BP1- and TIA1-positive stress granules in response to hyperosmolar shock and arsenite treatment in N2A cells. In summary, the aberrant mutant SOD1–G3BP1 interaction affects stress granule dynamics, suggesting a potential link between pathogenic SOD1 mutations and RNA metabolism alterations in ALS.

**Electronic supplementary material:**

The online version of this article (doi:10.1007/s00401-016-1601-x) contains supplementary material, which is available to authorized users.

## Introduction

Amyotrophic lateral sclerosis (ALS or Lou Gehrig’s disease) is a progressive neurodegenerative disease with no cure available [37, 49, 71]. A better understanding of the molecular etiology of the disease is needed to develop effective preventive measures or cures. Approximately 10–15 % of ALS cases are familial and studies of the ALS genes whose mutations cause familial ALS have provided valuable insights into the disease mechanism. The first identified ALS gene encodes copper/zinc superoxide dismutase (SOD1) [14, 51]. The ALS mutations in SOD1 cause toxicity that is foreign to the wild-type (WT) protein (termed as “gain-of-function”) [9], but the nature of such toxicity is still not fully understood. Mutations in a group of genes involved in RNA metabolism have been found in recent years, including TDP-43 [45], FUS [35, 67], ataxin-2 [19], hnRNPA1 [33] and Matrin-3 [29]. The seemingly unrelated pathogenic mechanisms elicited by the mutations in SOD1 and in RNA metabolism regulators have not been reconciled yet.

The Ras GTPase-activating protein-binding protein G3BP1 [48] is an important regulator of RNA metabolism [5, 24, 66], translation [1, 47] and stress granule (SG) dynamics [2, 65, 70]. G3BP1 was reported to play a critical role in the secondary aggregation step of SG formation [2], and has been used as a reliable marker of SGs [31]. The misregulation of SG dynamics has been reported in many forms of ALS [36]. G3BP1 is critical for neuronal survival since G3BP1 null mice demonstrate widespread neuronal cell death in the central nervous system [73]. G3BP1 is also critical for synaptic plasticity and calcium homeostasis [43].

In this study, we initially tested whether aggregated ALS mutant SOD1-containing inclusions were in any way related to G3BP1-positive stress granules. Interestingly, mutant SOD1 inclusions were co-localized with G3BP1-positive granules in spinal cord motor neurons of G93A SOD1 transgenic mice as well as in cultured cells. ALS-related mutants of SOD1, unlike wild-type SOD1, interacted with G3BP1 and the interaction was preserved in the presence of RNase, suggesting a direct protein–protein interaction. Domain deletion mutations, molecular modeling and point mutagenesis showed that the RNA-binding RRM domain of G3BP1 interacted with mutant SOD1 via residues critical to the RNA binding. Furthermore, the expression of mutant SOD1 perturbed SG dynamics and resulted in a delayed formation of SG in response to hyperosmolar stress and arsenite treatment. Our results suggest that G3BP1 represents a potential link between pathogenic SOD1 mutations and RNA metabolism alterations in ALS.

## Materials and methods

### Plasmids

The WT and A4V mutant SOD1-EGFP [74], SOD1-3xHA [21], 3xFLAG-FUS [22], FLAG-TDP-43 (a gift from Dr. Francisco Baralle) [3], FLAG-MATR3 (a gift from Dr. Yossi Shiloh, Addgene plasmid # 32880) [52], and FLAG-hnRNPA1 (a gift from Dr. J. Paul Taylor) [33] expression constructs were previously reported. The human G3BP1 expression constructs used in this study were based on the FLAG-G3BP1 plasmid [34], a generous gift from Dr. Zhi-Min Yuan (University of Texas Health Science Center at San Antonio). The 3xFLAG-tagged G3BP1 constructs were generated from p3xFLAG-CMV10 (Sigma) using standard cloning techniques. The F380L/F382L G3BP1 mutation and the W32S SOD1 mutation were introduced with the QuikChange II Site-Directed Mutagenesis Kit (Agilent). The A4V/W32S double mutant SOD1-EGFP and the EGFP-G3BP1 expression constructs were generated by subcloning the respective fragments to pEGFP-N3 and pEGFP-C3 (Clontech), respectively. The mCherry-WT and F380L/F382L double mutant G3BP1 constructs were made by subcloning the respective G3BP1 fragments to pmCherry-C1 (Clontech). All plasmid constructs were verified with sequencing.

### Cell culture and transfection

N2A and HEK293T (293T) cells were cultured in DMEM (Sigma, D5796) supplemented with 10 % fetal bovine serum and penicillin–streptomycin at 37 °C in 5 % CO_2_/95 % air with humidification. N2A and 293T cells were transfected with Lipofectamine 2000 (Life Technologies) and Polyethylenimine “Max” (Polysciences, Inc.), respectively. The G3BP1-null 293T cells were generated by employing CRISPR technology (G3BP1 Double Nickase Plasmids, Santa Cruz, sc-400745-NIC) following the manufacturer’s protocol.

### Animals

Transgenic mouse strains B6.Cg-Tg(SOD1)2Gur/J and B6.Cg-Tg(SOD1-G93A)1Gur/J [26] were bred and maintained as hemizygotes at the University of Kentucky animal facility. Transgenic mice were identified using PCR. The mice were killed at age 60, 90 and 125 ± 5 days. Mice were anesthetized with an intraperitoneal injection of 0.1 ml Pentobarbital (50 mg/ml, Abbott Laboratories) and transcardially perfused with 0.1 M phosphate buffered saline (PBS), pH 7.5 before spinal cords were dissected. All animal procedures were approved by the university IACUC committee.

### Clinical materials

Human skin fibroblast cell cultures were established as previously described [16]. Briefly, punch skin biopsy (3 mm) was obtained after informed consent from a 63-year-old male with symptomatic ALS with a documented L144F SOD1 mutation (Athena Diagnostics). The control skin biopsy was obtained from a 64-year-old healthy male who was free of neurological disease or any known ALS gene mutation. Skin biopsies were washed with PBS and cut into small pieces. Fibroblast growth medium [MEM (Sigma, M5650) supplemented with 20 % FBS, 2 mM l-glutamine, 100 unit/ml penicillin, and 100 µg/ml streptomycin] was added to the minced biopsy tissue and transferred along with tissue fragments into tissue culture plates. Cultures were maintained in a humidified atmosphere of 5 % CO_2_/95 % air at 37 °C to allow fibroblast cells grow from tissue fragments. Fibroblast cells were then maintained under the same conditions as above. The study was approved by the Institutional Review Board of the University of Kentucky.

### Fluorescence microscopy

N2A or 293T cells were seeded on gelatin-treated glass coverslips and transfected with SOD1-EGFP constructs. Twenty-four hours later, cells were rinsed with 1× PBS, fixed with 4 % formaldehyde in 1× PBS, and permeabilized with 1× PBS supplemented with 0.25 % Triton-X100. Primary fibroblast cells were cultured, fixed and permeabilized similarly as above. Mouse spinal cords were dissected, post-fixed in 4 % formaldehyde in 1× PBS for 3 h, cryopreserved in 30 % sucrose overnight, embedded in Tissue-Tek OCT compound (Sakura). Sections were cut at 12 μm and permeabilized with 1× PBS supplemented with 0.1 % Triton-X100. The primary antibodies were sheep anti-human SOD1 (The Binding Site, PC077), mouse anti-G3BP1 (BD Biosciences, 611126), rabbit anti-G3BP1 (Proteintech, 13057-2-AP), goat anti-TIA1 (Santa Cruz, sc-1751), mouse anti-eIF3 p110 (Santa Cruz, sc-74507) and mouse anti-GE-1/p70 S6 kinase alpha (Santa Cruz, sc-8418). The secondary antibodies were Alexa Fluor 488 donkey anti-sheep (Life Technologies, A11015), Alexa Fluor 568 donkey anti-mouse (Life Technologies, A10037), Alexa Fluor 647 donkey anti-mouse (Life Technologies, A-31571), Alexa Fluor 568 donkey anti-rabbit (Life Technologies, A-10042), Alexa Fluor 647 donkey anti-rabbit (Life Technologies, A-31573), and Alexa Fluor 568 donkey anti-goat (Life Technologies, A-11057). The samples were mounted by applying Vectashield Mounting Medium (Vector Laboratories) and visualized using a Nikon A1 or a Leica SP5 confocal microscope with a 60× objective.

### Co-immunoprecipitation assays

The lysates were prepared in 1× RIPA buffer (Millipore) supplemented with protease inhibitor cocktail (Sigma, P-8340, 1:500) and 1 mM sodium orthovanadate. Mouse spinal cord extracts were prepared using a dounce homogenizer. Transfected cell lysates were homogenized by passing through a 23-gauge needle several times. Immunoprecipitations were performed at 4 °C for 2 h. The endogenous G3BP1 immunoprecipitations were done from spinal cord extracts with rabbit anti-G3BP1 Antibody (Millipore, 07-1801) and Protein A UltraLink Resin (Thermo Scientific Pierce, 53139). The FLAG immunoprecipitations were performed using EZview Red Anti-FLAG M2 Affinity Gel (Sigma, F2426) and the bound proteins were eluted with 3xFLAG peptide (Sigma, F4799). Where indicated, RNase Cocktail (Ambion Life Technologies, AM2286) was added to the immunoprecipitation mixtures at 1:100 dilution.

The in vitro SOD1–G3BP1 binding assays were performed using 2 μg WT or G93A mutant human SOD1 purified from insect cells as described [27] and 1.5 μg of 6xHis-tagged human G3BP1 purified from *E. coli* (Fitzgerald Industries, 80R-1601) in 500 μl 1× RIPA buffer. The mixtures were incubated at 37 °C for 2 h, cooled to 4 °C and G3BP1 was immunoprecipitated with mouse anti-G3BP1 (Millipore, 05-1938) and Protein G UltraLink Resin (Thermo Scientific Pierce, 53126).

### Western blotting

The nitrocellulose membranes were blocked and antibodies were applied in 5 % milk in TBST (100 mM TRIS–HCl, pH7.5, 0.9 % NaCl, 0.1 % Tween-20). The antibodies used were rabbit anti-G3BP1 (Millipore, 07-1801), rabbit anti-SOD1 (Santa Cruz, sc-11407), mouse anti-FLAG M2-HRP (Sigma, A8592), rabbit anti-HA (Santa Cruz, sc-805), mouse anti-PABP1 (Santa Cruz, sc-32318) and goat anti-actin (Santa Cruz, sc-1616). All immunoblotting images were acquired using a BioRad ChemiDoc MP system.

### In silico docking

The homology model of the RRM domain of G3BP1 was obtained using a leading homology modeling server Raptor X [30]. The model was docked to the SOD1 A4V mutant dimer (PDB ID: 3GZQ, [23]) using the protein–protein interaction modeling server HADDOCK [17]. The docking model was further refined using Rosetta Docking [12] as implemented in the ROSIE server [40], which evaluates protein–protein complexes by using rigid body perturbations of the protein chains.

### Stress granule induction and analysis

N2A cells grown on gelatin-treated glass coverslips were transfected with WT or A4V mutant SOD1–EGFP constructs. Twenty-four hours later, the cells were treated with 0.5 M d-sorbitol (Sigma, S1876) or 0.5 mM sodium arsenite dissolved in fresh medium for the indicated times at 37 °C to induce stress granules, with or without recovery in fresh medium as indicated. Control cells were treated with fresh medium without sorbitol or arsenite. The cells were fixed and G3BP1 immunofluorescence experiments were performed as above. Z-stack images of random view-fields were acquired with identical imaging parameters. Maximum intensity projections of the Z-stacks were analyzed for stress granule formation as published [10] using ImageJ (http://imagej.nih.gov/ij). ANOVA with post hoc Tukey HSD (honest significant difference) test was used to determine *p* values for multiple pair-wise comparisons. Student’s *t* test (two-tailed distribution, two-sample unequal variance) was used to determine *p* values for simple pair-wise comparison.

## Results

### Mutant SOD1 inclusions are co-localized with stress granule markers in ALS model systems

**Fig. 1:**
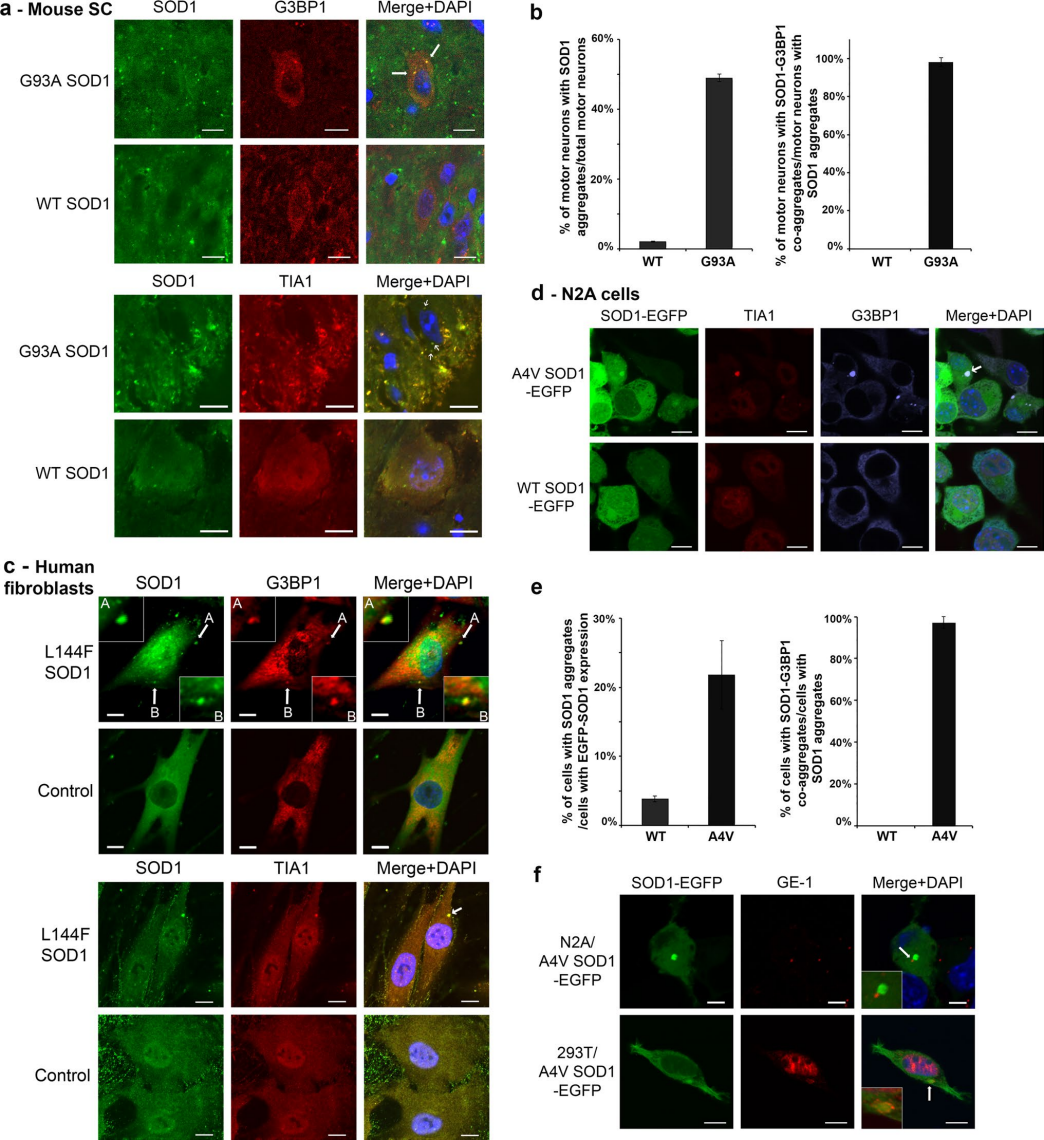
G3BP1 co-localizes with ALS mutant SOD1 inclusions. **a** Immunofluorescence of SOD1, G3BP1 and TIA1 in the spinal cord of 90-day-old G93A and WT SOD1 transgenic mice. *Top* G3BP1 and G93A mutant SOD1 are co-localized in inclusions in motor neurons (illustrated by *arrows*) while WT SOD1 shows even cytoplasmic distribution. *Bottom* TIA1 and G93A mutant SOD1 are co-localized in inclusions in motor neurons (illustrated by *arrows*). **b** Quantification of G93A SOD1 inclusions and the co-localization of G93A SOD1 and G3BP1 in mouse spinal motor neurons. Three pairs of WT and G93A mice were examined and a total of over 200 motor neurons were counted. **c** Immunofluorescence of SOD1, G3BP1 and TIA1 in human fibroblasts harboring the L144F ALS mutation in SOD1 and in control fibroblasts. *Top* G3BP1 and SOD1 are co-localized in inclusions in the L144F SOD1 ALS patient-derived cells (illustrated by *arrows*), whereas WT SOD1 shows even cytoplasmic distribution in the control cells. *Bottom* TIA1 and L144F mutant SOD1 are co-localized in inclusions as illustrated by the *arrow*. **d** Confocal microscopic images of G3BP1, TIA1 and SOD1 in N2A cells expressing A4V mutant (*top*) or WT (*bottom*) SOD1. A4V mutant SOD1, G3BP1 and TIA1 are co-localized in inclusions with the size of approximately 1–3 µm, whereas WT SOD1 shows even cytoplasmic distribution. **e** Quantification of A4V SOD1 inclusions and the co-localization of A4V SOD1 and G3BP1 in N2A cells. Three independent experiments were performed and at least five random viewfields from each experiment were included in the quantification. **f** Confocal microscopic images of A4V mutant SOD1 and the P-body marker GE-1 in N2A (*top*) and 293T (*bottom*) cells. Mutant SOD1 inclusions and P-bodies are illustrated by *arrows* and zoom-in images show that they are localized in close proximity to each other. The confocal microscopic images were acquired with a Nikon A1 microscope. The nuclei were stained with DAPI. *Scale bars* 10 μm

We set forth to determine whether there is a correlation between G3BP1-positive stress granules and mutant SOD1 inclusions in murine and cellular ALS model systems. Interestingly, we found that mutant SOD1 inclusions generally co-localized with G3BP1 in spinal cord motor neurons from 90-day-old symptomatic G93A mutant SOD1 transgenic mice (Fig. 1a). In contrast, no obvious G3BP1 or SOD1 inclusions were observed in age- and gender-matched WT SOD1 transgenic mice. The G93A SOD1 inclusions also co-localized with an additional stress granule marker, TIA1 [31] (Fig. 1a). The co-localization of all three proteins, G93A SOD1, TIA1 and G3BP1 was also demonstrated (Supplemental Fig. 1). No fluorescence signals were observed in primary antibody-omitted samples (Supplemental Fig. 2). Examination of three pairs of WT and G93A SOD1 mice showed that 49 % spinal cord motor neurons in 90-day-old G93A mice contained SOD1 inclusions and nearly all motor neurons with SOD1 inclusions also contained G3BP1 co-inclusions (Fig. 1b). In contrast, less than 3 % motor neurons in WT SOD mice showed SOD1 inclusions and no WT SOD1–G3BP1 co-inclusions were observed.

In addition, the co-localization of G3BP1 with SOD1 inclusions was observed in human fibroblasts derived from an ALS patient carrying the L144F SOD1 mutation (Fig. 1c). The SOD1 inclusions were also co-localized with TIA1 in L144F SOD1 ALS patient fibroblast cells (Fig. 1c, Supplemental Fig. 3). In contrast, no SOD1 inclusions were observed in fibroblast cells derived from an age- and gender-matched control (Fig. 1c, Supplemental Fig. 3).

In N2A cells transfected with A4V mutant SOD1, the G3BP1-positive mutant SOD1 inclusions were mostly round shaped with approximately 1–3 μm diameter (Fig. 1d). Quantification of 20 randomly selected view-fields from three independent experiments showed that approximately 22 % of transfected cells contained mutant SOD1 inclusions, and 97 % of N2A cells with mutant SOD1 inclusions also showed G3BP1 co-inclusions (Fig. 1d). Approximately 4 % of N2A cells expressing WT SOD1 contained inclusions and no G3BP1 co-inclusions were observed. The mutant SOD1 inclusions also co-localized with TIA1 (Fig. 1d) and another SG marker eIF3 [31] (Supplemental Fig. 4) in N2A cells. Similar co-localization of mutant SOD1 inclusions and G3BP1 was observed in transfected 293T cells (Supplemental Fig. 5).

Processing bodies (P-bodies) are discrete cytoplasmic foci where mRNA degradation takes place [55] and are often intimately juxtaposed to stress granules [32]. We found that mutant SOD1 inclusions in N2A and 293T cells did not stain positive for the P-bodies marker GE-1 [31, 57], but were in close proximity to GE-1 positive P-bodies instead (Fig. 1f). The results collectively suggest that mutant SOD1 inclusions, which are positive for multiple stress granule markers, are likely stress granules.

### ALS mutants of SOD1 interact with G3BP1

**Fig. 2:**
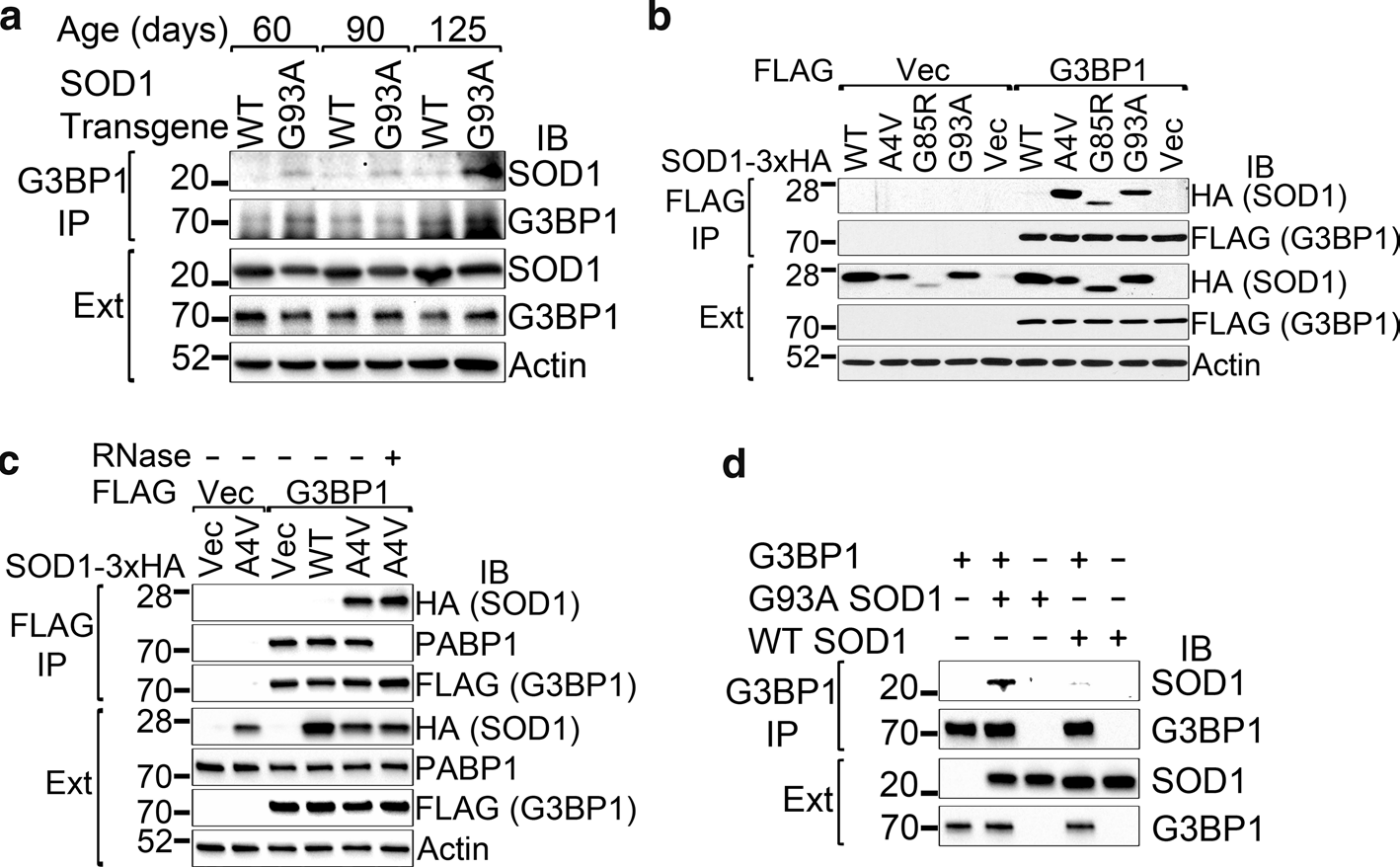
ALS mutants of SOD1 interact with G3BP1. **a** Endogenous G3BP1 was immunoprecipitated from 60, 90 or 125-day-old WT or G93A human SOD1 transgenic mouse spinal cord extracts. A significantly higher amount of G93A SOD1 was co-precipitated with G3BP1 than WT SOD1 was. **b** FLAG-G3BP1 was immunoprecipitated from cellular extracts of 293T cells transfected with the indicated expression constructs. Multiple ALS-causing mutants were co-precipitated with G3BP1. **c** FLAG-G3BP1 was immunoprecipitated from cellular extracts of 293T cells transfected with the indicated expression constructs, with or without the inclusion of RNase in the immunoprecipitation, as indicated. **d** G3BP1 purified from *E. coli* and WT or G93A mutant SOD1 purified from insect cells were combined in vitro, followed by G3BP1 immunoprecipitation. The immunoprecipitations were followed by western blotting with the indicated antibodies. The positions and sizes of the closest molecular weight marker bands are shown. *IB* immunoblot, *IP* immunoprecipitation, *Ext* whole cell extracts (**a**–**c**) or pre-IP mixture (**d**)

G3BP1 immunoprecipitation was performed with spinal cord extracts from 60-, 90- and 125-day-old WT or G93A mutant SOD1 transgenic mice. A small amount of WT SOD1 was co-precipitated with G3BP1 while a significantly higher amount of G93A mutant SOD1 was co-precipitated with G3BP1 (Fig. 2a). We next tested whether G3BP1 can interact with other ALS-related SOD1 mutants. Cells were co-transfected with 3xHA-tagged WT or A4V, G85R or G93A mutant SOD1 and FLAG-tagged G3BP1 or FLAG vector control. FLAG immunoprecipitation showed that A4V, G85R and G93A mutant SOD1 all co-precipitated with FLAG-G3BP1 (Fig. 2b, lanes 7–9) while no co-precipitation of WT SOD1 was detected (lane 6). As negative controls, no WT or mutant SOD1 was co-precipitated with the FLAG vector (Fig. 2b, lanes 1–5).

### ALS mutant SOD1 directly interacts with G3BP1

Since G3BP1 is an RNA-binding protein, we tested whether the mutant SOD1–G3BP1 interaction was dependent on RNA. RNase was either included or omitted in the immunoprecipitation mixture and the FLAG-G3BP1 co-immunoprecipitation experiment was subsequently performed. Mutant SOD1 was still co-precipitated by G3BP1 in the presence of RNase (Fig. 2c, lanes 5, 6). The poly A-binding protein PABP1 was included as a control of the RNase treatment since G3BP1 was reported to interact with PABP1 in an RNA-dependent manner [44, 58]. The interaction of G3BP1 with the endogenous PABP1 was completely abolished in the presence of RNase (Fig. 2c, lanes 5, 6). These results support that the G3BP1–mutant SOD1 interaction was RNA-independent.

We next tested whether the G3BP1–mutant SOD1 interaction was direct. Purified WT or G93A mutant SOD1 and purified G3BP1 were used for immunoprecipitation. G93A SOD1, unlike WT SOD1, efficiently co-precipitated with G3BP1 (Fig. 2d, lane 2 versus lane 4), showing that mutant SOD1 and G3BP1 interact directly.

### The specificity of the mutant SOD1–G3BP1 interaction

**Fig. 3:**
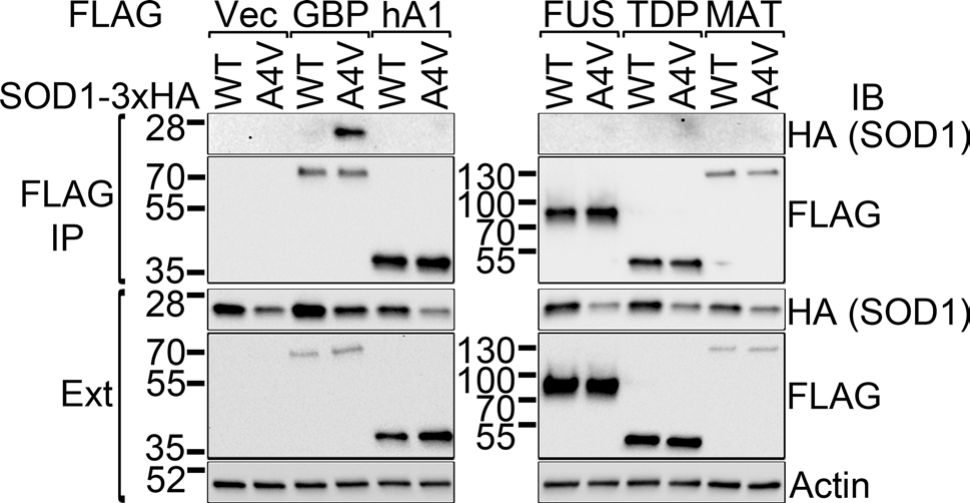
Mutant SOD1 selectively interacts with G3BP1. FLAG-tagged G3BP1 (GBP), hnRNPA1 (hA1), FUS, TDP-43 (TDP) or Matrin-3 (MAT) was co-transfected with HA-tagged WT or A4V SOD1. FLAG immunoprecipitations were performed and followed by western blotting with the indicated antibodies. A4V SOD1 was only co-precipitated with G3BP1 but not other RNA-binding proteins. *IB* immunoblot, *IP* immunoprecipitation, *Ext* whole cell extracts

We next tested whether mutant SOD1 interacts with other RRM domain-containing RNA-binding proteins or specifically with G3BP1. The RNA-binding proteins implicated in ALS, hnRNPA1, FUS, TDP-43 and Matrin-3 were examined. Cells were co-transfected with WT or A4V mutant SOD1 along with FLAG-tagged G3BP1, hnRNPA1, FUS, TDP-43 or Matrin-3. FLAG immunoprecipitation was performed and A4V mutant SOD1 only co-precipitated with G3BP1 (Fig. 3, lane 4), but not with any of the other four FLAG-tagged RNA-binding proteins (hnRNPA1, FUS, TDP-43 and Matrin-3).

### Mutant SOD1 interacts with the RRM domain of G3BP1 via residues critical to RNA binding

**Fig. 4:**
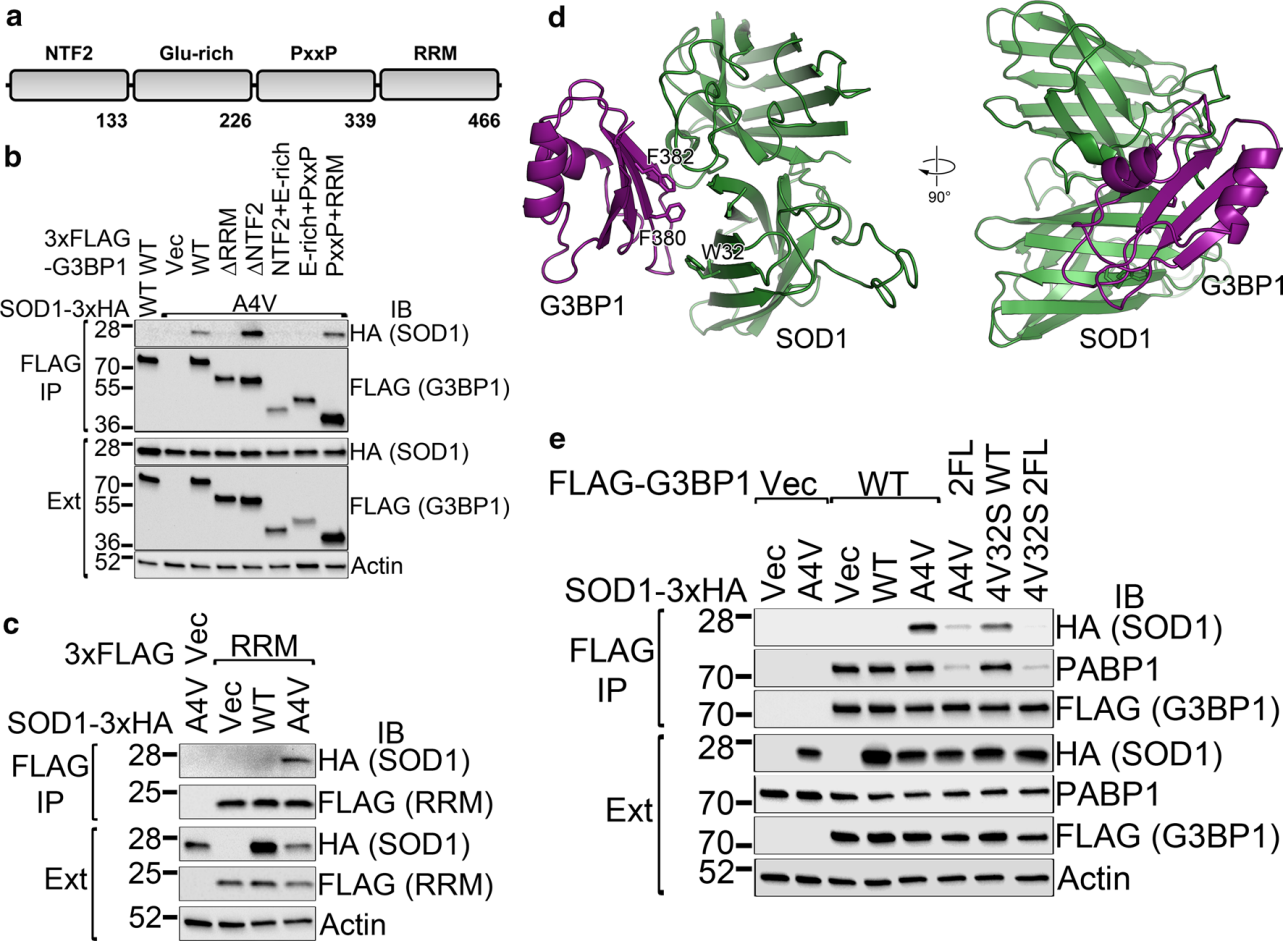
The binding of mutant SOD1 is mediated by the RRM domain of G3BP1. **a** The schematic domain structure of G3BP1. **b** The RRM domain of G3BP1 is necessary for the binding of mutant SOD1. A series of domain deletion mutants of G3BP1 were tested in FLAG-G3BP1 immunoprecipitations. **c** The isolated RRM domain of G3BP1 is sufficient for binding to mutant SOD1. FLAG immunoprecipitations were performed on cellular extracts with the expression of indicated constructs. **d** In silico docking of A4V mutant SOD1 (*green*) and the RRM domain of G3BP1 (*purple*). The F380 and F382 residues of G3BP1 and W32 of SOD1 are shown in *stick representation*. **e** The F380 and F382 residues of G3BP1 and the W32 residue of mutant SOD1 are important for the mutant SOD1–G3BP1 interaction. FLAG immunoprecipitations were performed followed by western blotting with the indicated antibodies. *2FL* F380L/F382L double G3BP1 mutant, *4V32S* A4V/W32S double SOD1 mutant, *IB* immunoblot, *IP* immunoprecipitation, *Ext* whole cell extracts

G3BP1 is a multi-domain protein consisting of an NTF2 oligomerization domain, a glutamic acid-rich domain, a PxxP domain and an RNA-binding RRM domain [65] (Fig. 4a). To determine which domain is responsible for the binding of mutant SOD1, we generated G3BP1 domain truncation mutants and tested their ability to co-precipitate A4V SOD1. Removing the RRM domain abolished the co-precipitation of A4V mutant SOD1 (Fig. 4b, lane 4), whereas truncation of the NTF2 domain did not impair A4V SOD1 binding (lane 5). In addition, the PxxP-RRM domain fragment co-precipitated with A4V mutant SOD1 (lane 8), whereas the NTF2-Glu-rich domain fragment (lane 6) or the Glu-rich domain–PxxP domain fragment (lane 7) did not co-precipitate with mutant SOD1. Furthermore, the RRM domain alone also co-precipitated with A4V SOD1 (Fig. 4c). Thus, the RRM domain of G3BP1 is necessary and sufficient for mutant SOD1 binding.

The ALS-related mutants of SOD1 were reported to display higher hydrophobicity than WT SOD1 [64]. We hypothesized that a hydrophobic gain-of-interaction might be responsible for the mutant SOD1–G3BP1 interaction. In the lack of an experimentally determined structure of G3BP1, a homology model of the RRM domain of G3BP1 was obtained with a *p* value 4.0 × 10^−6^ based on Bruno protein RRM domain (PDB ID: 2KHC) as the template using a leading homology modeling server Raptor X [30]. We next performed in silico docking of the G3BP1 RRM homology model with the crystal structure of A4V mutant SOD1 (PDB ID: 3GZQ, [23]) using the HADDOCK protein–protein interaction modeling server [17]. The docking model was further refined using the Rosetta Docking algorithm [12] that evaluates protein–protein complexes by using rigid body perturbations of the protein chains. The docking model with the minimal energy is shown in Fig. 4d. The model positioned the RRM domain of G3BP1 above the crevice between two monomers of SOD1. Interestingly, the F380 and F382 residues of G3BP1 RRM and the W32 residue of SOD1 are located in close proximity (Fig. 4d), suggesting that these residues are critical to the interaction.

We generated the F380L/F382L double mutation (2FL), which caused a significant loss of interaction with mutant SOD1 (Fig. 4e, lane 6 compared to lane 5). In addition, we introduced a W32S mutation into A4V SOD1 and found that the co-precipitation of the A4V/W32S mutant with G3BP1 was impaired as compared to the A4V mutant (lane 5 versus lane 7). The interaction between A4V/W32S SOD1 and F380L/F382L G3BP1 was barely detectable (lane 8) as compared to the interaction between A4V SOD1 and wild-type G3BP1 (lane 5). Therefore, the F380 and F382 residues in the RRM domain of G3BP1 and the W32 residue of SOD1 directly participate in the mutant SOD1–G3BP1 interaction.

### The mutant SOD1–G3BP1 interaction is critical to the co-localized inclusions

**Fig. 5:**
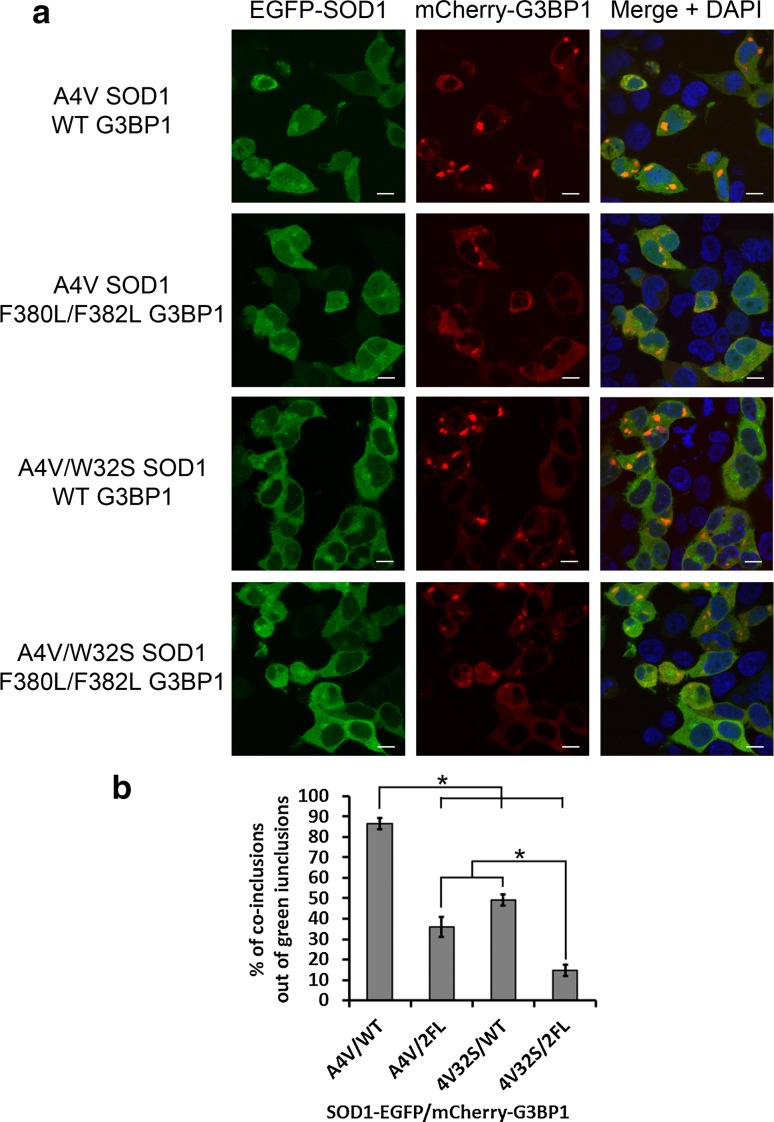
The effect of W32 residue in SOD1 and F380 and F382 residues in G3BP1 on co-inclusions. **a** Confocal microscopic images of G3BP1-null 293T cells transfected with combinations of EGFP-tagged A4V or A4V/W32S SOD1 and mCherry-tagged WT or F380L/F382L mutant G3BP1. **B** More than 150 cells in 10 random view-fields were quantified as with *green*, *red* and *green*/*red* co-localized inclusions. The percentage of cells with *green*/*red* co-inclusions versus the total number of cells with *green* inclusions was calculated. The results of three independent parallel experiments are shown. Mutations of W32, F380 and F382 residues in individual or both proteins reduced the co-inclusions of mutant SOD1 and G3BP1. *p values less than 0.01 as determined by ANOVA with post hoc Tukey HSD test

We tested the significance of mutant SOD1–G3BP1 interaction in the formation of protein inclusions by mutating the W32 residue in SOD1 and the F380 and F382 residues in G3BP1. G3BP1-null 293T cells were transfected with combinations of EGFP-tagged A4V or A4V/W32S double mutant SOD1 and mCherry-tagged WT or F380L/F382L double mutant G3BP1 (Fig. 5a). The number of transfected cells with green, red and co-localized green/red inclusions was counted from more than 150 cells in ten random view-fields. The percentage of co-localized green/red inclusions out of the total number of green inclusions in the above groups of cells is shown in Fig. 5b. Whereas A4V SOD1 co-localized with WT G3BP1 in 86.6 % of cells with SOD1 inclusions, the F380L/F382L G3BP1 mutation significantly decreased the co-localization to 36.1 % (*p* = 0.001), and the W32S SOD1 mutation to 49.1 % (*p* = 0.001). The combination of the W32S SOD1 mutation and the F380L/F382L G3BP1 mutation resulted in only 14.7 % of cells with SOD1 inclusions with co-localizing G3BP1 (*p* = 0.001) (Fig. 5b). The *p* value of comparing the single protein mutation, i.e., F380L/F382L G3BP1 mutation or W32S SOD1 mutation, to the combined mutations in both proteins is also 0.001. The *p* values for the above pair-wise comparisons were determined by ANOVA with post hoc Tukey HSD test. The results suggest that, disruption of the mutant SOD1–G3BP1 interaction by substituting the critical residues (W32 in SOD1 and F380 and F382 in G3BP1) significantly reduced the double-positive inclusions.

### Mutant SOD1 interferes with the dynamics of induced G3BP1 stress granules

**Fig. 6:**
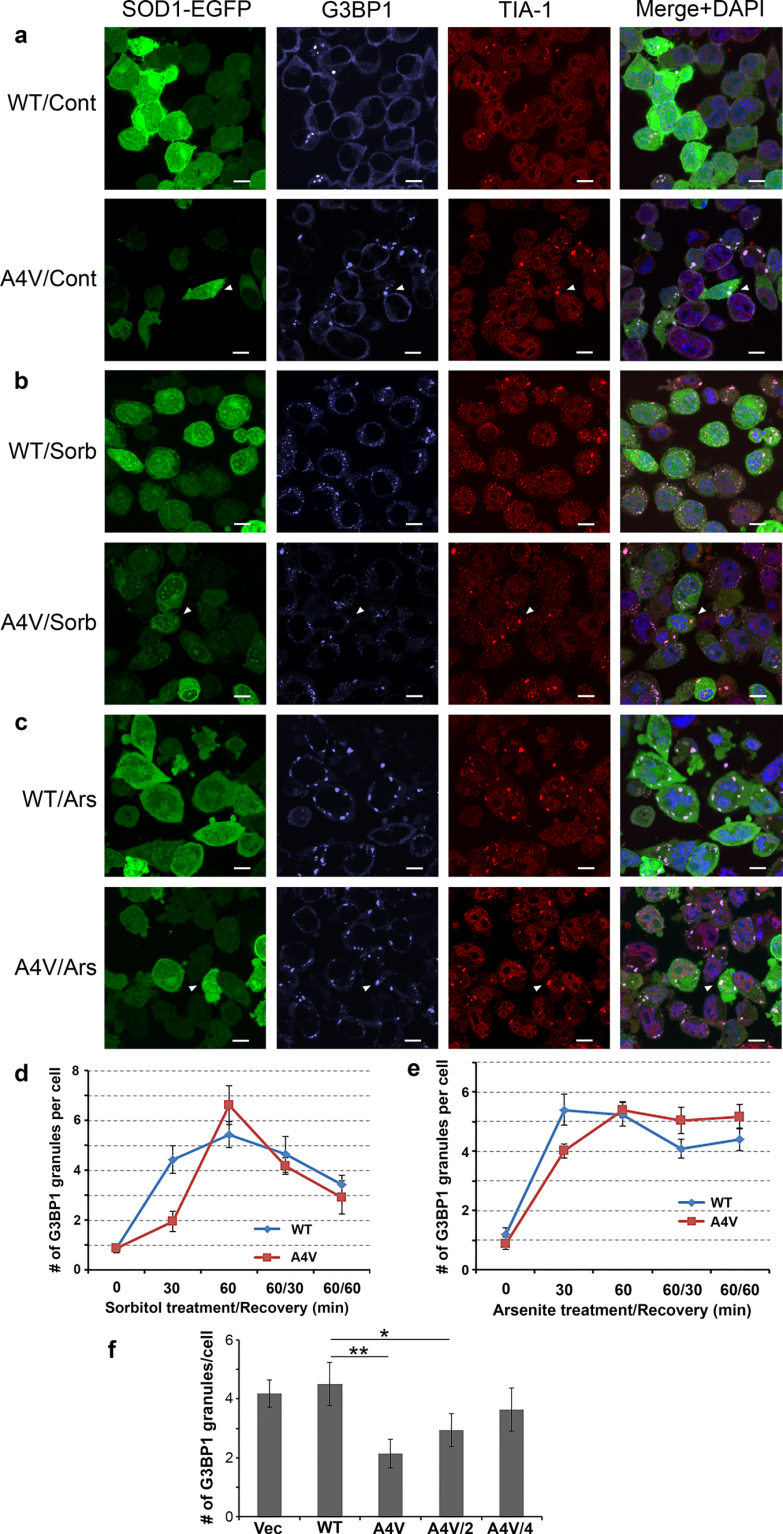
Mutant SOD1 expression interferes with the induction of G3BP1 stress granules upon hyperosmolar stress or arsenite treatment. Confocal microscopic images of N2A cells expressing EGFP-tagged WT or A4V SOD1 (*green*) without treatment (**a**), or 30 min of sorbitol-induced hyperosmolar stress (**b**), or 30 min of arsenite treatment (**c**). The endogenous G3BP1 (*purple*) and TIA1 (*red*) were stained and images were acquired with a Nikon A1 microscope. The nuclei were stained with DAPI. *Scale bars* 10 μm. **d** Time course of the number of G3BP1 granules upon sorbitol-induced hyperosmolar stress, with or without recovery in fresh medium. **e** Time course of the number of G3BP1 granules upon arsenite treatment, with or without recovery in fresh medium. **f** Quantification of the number of G3BP1 granules in N2A cells transfected with EGFP vector or EGFP-tagged WT or A4V SOD1. A half or a quarter amount of A4V plasmid was used to examine how A4V mutant SOD1 levels influenced the number of G3BP1-positive granules. *p  < 0.05 and **p  < 0.01 as determined by ANOVA with post hoc Tukey HSD test. The quantification was based on four parallel experiments, representing over 250 randomly selected cells for each cell population

We hypothesized that the interaction of mutant SOD1 with G3BP1 could perturb the G3BP1-mediated stress granule dynamics. We tested this hypothesis by examining the formation of induced G3BP1 stress granules in N2A cells transfected with WT or A4V mutant SOD1 upon exposure to hyperosmolar stress (Fig. 6b) or arsenite treatment (Fig. 6c). The cells were treated with 0.5 M sorbitol or 0.5 mM arsenite dissolved in fresh medium for 30 or 60 min, or allowed to recover for 30 or 60 min in fresh medium after 60 min of stress. Control cells were subjected to the same procedure with medium not containing sorbitol or arsenite (Fig. 6a). The cellular distributions of SOD1-EGFP, G3BP1 (purple) and an additional stress granule marker TIA1 (red) were determined by immunofluorescence (Fig. 6a–c). It is noted that nearly all G3BP1 granules were also positive of TIA1.

The number of G3BP1 granules per cell was quantified from ten random view-fields for each cell population at each time point. We found that after 30 min of hyperosmolar stress, the average number of G3BP1 granules was significantly less in cells expressing A4V mutant SOD1 as compared to cells expressing WT SOD1 (*p* = 0.005, Student’s *t* test) (Fig. 6d), suggesting a delayed formation of stress granules in response to hyperosmolar stress. Similarly, the average number of G3BP1 granules was significantly less in cells expressing A4V mutant SOD1 as compared to cells expressing WT SOD1 (*p* = 0.01, Student’s *t* test) (Fig. 6e) 30 min after arsenite treatment. The results consistently suggest that mutant SOD1 delayed the formation of stress granules.

We further tested the effect of different levels of A4V expression on the formation of G3BP1-positive stress granules (Fig. 6f). N2A cells were transfected with EGFP vector control or EGFP-tagged WT, A4V, or only half or quarter of A4V plasmids. The total amount of plasmids used in all experiments was constant by including EGFP vector as make-up. There was no statistically significant difference in the number of G3BP1 granules in cells transfected with the EGFP vector control or WT SOD1, suggesting that the EGFP tag did not interfere with the experiment. Transfection of the full or half amount of A4V SOD1 resulted in statistically significant reduction of the number of G3BP1 granules as compared to WT SOD1 transfection (*p* = 0.001 and 0.03, respectively, ANOVA with post hoc Tukey HSD test), whereas transfecting only the quarter amount of A4V SOD1 did not result in statistically different number of G3BP1 granules. Accordingly, our results support the hypothesis that mutant SOD1 affects the dynamics of G3BP1-positive stress granules, specifically delayed the formation of stress granules.

## Discussion

SOD1 was the first gene whose mutations were identified to cause familial ALS [14, 51]. The cytoplasmic SOD1-positive protein inclusions in affected motor neurons are a hallmark of mutant SOD1-mediated ALS [50, 68]. In other ALS cases, the pathological inclusions are often immune-positive of the RNA-binding protein TDP-43 [41, 60]. In recent years, alterations in the dynamics of stress granules have emerged as a common theme in a wide range of ALS cases [18, 36, 72]. ALS-related mutations in a range of genes including TDP-43, FUS, hnRNPA1, profilin-1, angiogenin and C9ORF72 were found to result in abnormalities in stress granule dynamics [2, 4, 7, 13, 20, 22, 33, 38, 59, 61, 63, 69]. Ataxin-2, an ALS risk factor with polyglutamine expansions, can also regulate stress granules [19, 46]. These observations suggest that mutant SOD1-mediated ALS might represent an outlier in the ALS disease mechanism. At the same time, the nature of the gain-of-toxicity caused by mutant SOD1 [9] is still not fully understood. This study was initiated to determine whether mutant SOD1 also causes perturbed stress granule dynamics.

We have demonstrated here that cytoplasmic inclusions of mutant SOD1 were immune-positive for two reliable stress granule markers G3BP1 [31, 32] and TIA1 in the G93A mutant SOD1 transgenic mouse spinal cord (Fig. 1a, Supplemental Fig. 1), fibroblast cells derived from human ALS patient (Fig. 1c, Supplemental Fig. 3), and cultured cells (Fig. 1d, Supplemental Figs. 4, 5). A third stress granule marker eIF3 was also co-localized with mutant SOD1 and G3BP1 in cultured N2A cells (Supplemental Fig. 4). Moreover, the G3BP1-positive mutant SOD1 inclusions were found closely juxtaposed to P-bodies (Fig. 1f), supporting that they constitute stress granules [32]. It was previously reported that RNA stabilizer Hu Antigen R (HuR) and TIA1-related protein (TIAR) were found along with mutant SOD1 in detergent-insoluble aggregates [39]. However, that study did not test whether mutant SOD1 was co-localized in stress granules. Our results provide the evidence that mutant SOD1 is co-localized with stress granules and consequently can potentially influence the dynamics of stress granules.

We found that the molecular basis for the co-localization of mutant SOD1 inclusions and G3BP1 is that multiple mutants of SOD1, but not WT SOD1, interact with G3BP1 (Fig. 2). This interaction was independent of RNA (Fig. 2c) and was reconstituted with purified mutant SOD1 and G3BP1 proteins (Fig. 2d), showing that mutant SOD1 and G3BP1 directly interact with each other. In the previous study showing the presence of HuR and TIAR in mutant SOD1 aggregates, the amount of HuR and TIAR was significantly reduced by RNase treatment [39], suggesting an indirect interaction between mutant SOD1 and HuR/TIAR. This is in contrast to the RNA-independent direct interaction between mutant SOD1 and G3BP1 found in this study. Moreover, the interaction appeared to be specific for G3BP1, while four other RNA-binding proteins implicated in ALS, hnRNPA1, FUS, TDP-43 or Matrin-3 did not interact with mutant SOD1 (Fig. 3).

We further demonstrated that the RRM domain of G3BP1 is essential and sufficient for the interaction with mutant SOD1 (Fig. 4b, c). Molecular modeling and in silico docking results suggest that the F380 and F382 residues of G3BP1 and the W32 residue of mutant SOD1 play a role in this interaction (Fig. 4d). Site-directed mutagenesis results showed that mutating F380 and F382 in G3BP1 and W32 in SOD1 indeed impaired the interaction (Fig. 4e). The W32 residue of human SOD1 resides in the middle of the third β-strand [25] and is exposed on the protein surface, thus it is reasonable that it is involved in the interaction with G3BP1. It has been reported that W32 potentiated the aggregation and cytotoxicity of ALS mutant SOD1 [62]. In addition, other ALS-related mutants of SOD1 were reported to display aberrantly increased hydrophobicity [64], which can enable the interaction with G3BP1. Consistent with the interaction results, the W32S SOD1 mutation and the F380L/F382L G3BP1 mutation significantly impaired the co-localization of A4V SOD1 with G3BP1 (Fig. 5).

We found that expression of mutant SOD1 delayed the formation of G3BP1 stress granules in response to hyperosmolar stress and arsenite treatment (Fig. 6). Hyperosmolar stress was chosen in our study since it was shown to induce FUS [53] and TDP-43 [15] stress granules. Arsenite treatment is widely used to induce stress granules as it induced oxidative stress [56]. There are multiple potential mechanisms how the ALS mutant SOD1–G3BP1 interaction could perturb stress granule dynamics. The F380 and F382 residues are well conserved in the “3” and “5” positions of the RNP1 motif (Supplemental Fig. 6A), which are critical to RNA binding [42]. Substitution of conserved phenylalanine residues in the RNP1 motif were reported to impair RNA binding [8, 11, 54]. It is noted that F380L/F382L mutation significantly reduced the interaction between G3BP1 and PABP1 (Fig. 4e, lanes 6 and 8). Similarly, the G3BP1-PABP1 interaction was abolished in the presence of RNase (Fig. 2c, lane 6), suggesting that F380L/F382L mutation impaired the RNA binding of G3BP1. Furthermore, we performed the RNA immunoprecipitation experiment to measure the amount of c-myc RNA (one of the few known RNA-binding partners of G3BP1 [66]) co-precipitated with G3BP1. The F380L/F382L double mutant G3BP1 co-precipitated significantly less endogenous c-myc mRNA than WT G3BP1 (*p* = 2.7 × 10^−4^), confirming that the F380 and F382 residues are important for G3BP1 RNA binding (Supplemental Fig. 6b). As discussed earlier, the interaction between mutant SOD1 and G3BP1 is not mediated by RNA, but rather direct protein–protein interaction (Figs. 2, 4). It is conceivable that, when F380 and F382 are engaged in the interaction with mutant SOD1, the RNA-binding ability of G3BP1 is impaired (a “competitive binding” model). Since G3BP1 plays a critical role in stress granule dynamics [2, 65, 70], its impaired RNA binding can result in the delayed formation of stress granules.

An alternative mechanism is that mutant SOD1 can sequester G3BP1 into inclusions, thus interfering with the G3BP1-mediated stress granule formation and dynamics. It is interesting to note “rimming” of mutant SOD1 inclusions by G3BP1 in cultured cells (Supplemental Fig. 5). Rimming of pathological aggregate structures was reported in other neurodegenerative diseases, e.g., the rimming of huntingtin aggregates by p62 [6] or HDAC6 [28]. This observation suggests that G3BP1 could be secondarily sequestered to mutant SOD1 inclusions, thus contributing to impaired stress granule dynamics (a “sequestration” model). Future studies are needed to distinguish these two potential mechanisms. It is also noted that G3BP1-positive granules were relatively small in cells under hyperosmolar stress (Fig. 6b) and large in cells treated with arsenite (Fig. 6c). In addition to the number of granules in this study, future studies using live cell imaging techniques will examine sizes of individual granules and determine whether multiple smaller granules merge into large granules under different stress conditions.

A delayed formation of stress granules in the presence of mutant SOD1 (Fig. 6) are reminiscent of the reports that the expression of ALS mutants of TDP-43 and FUS also resulted in altered stress granules [4, 15, 38]. These findings suggest that the misregulation of stress granule dynamics represents a common pathophysiological feature shared by ALS cases mediated by mutant SOD1 and those caused by mutations in proteins involved in RNA metabolism. Perturbation of the physiological function of G3BP1 is especially harmful to neurons since G3BP1 is a neuronal survival factor [43, 73]. The full complement of RNAs regulated by G3BP1 and in general by stress granules in the central nervous system is unknown. In addition, it remains unclear whether RNA molecules reside in the mutant SOD1 and G3BP1 positive co-inclusions or what functional role RNAs play in the inclusions. Future studies will determine the range of RNAs whose stability and function is affected by altered stress granule dynamics in ALS.

Taken together, our results suggest that the aberrant interaction of ALS-related SOD1 mutants with G3BP1 and the resulting perturbation of stress granule dynamics are likely important components of the toxicity of SOD1 mutations. In addition, a number of stress conditions are known to induce G3BP1-positive stress granules that might in turn seed mutant SOD1 inclusions. This two-hit scenario might enhance the pathological aggregation of mutant SOD1. Our findings reconcile the seemingly disparate disease mechanisms caused by mutations in SOD1 and several other genes involved in RNA metabolism.

## Electronic supplementary material

Below is the link to the electronic supplementary material.

Supplementary material 1 (PDF 366 kb)
