# Supplementary material for: ALS mutant SOD1 interacts with G3BP1 and affects stress granule dynamics
Source: Acta Neuropathol. 2016 Aug 1;132(4):563–76. doi: 10.1007/s00401-016-1601-x (PMC5023729; doi:10.1007/s00401-016-1601-x)

Supplemental Materials

ALS Mutant SOD1 Interacts with G3BP1 and Affects Stress Granule Dynamics

Jozsef Gal[1, §, *], Lisha Kuang[1, §], Kelly R. Barnett[1], Brian Z. Zhu[1, 2], Susannah C. Shissler[1], Konstantin V. Korotkov[1], Lawrence J. Hayward[3], Edward J. Kasarskis[4] and Haining Zhu[1, 5, #]

[1] Department of Molecular and Cellular Biochemistry, University of Kentucky, Lexington, KY 40536; [2] Math Science Technology Center, P L Dunbar High School, Lexington, KY 40513; [3] Department of Neurology, University of Massachusetts Medical School, Worcester, MA 01655; [4] Department of Neurology, University of Kentucky, Lexington, KY 40536; [5] Lexington VA Medical Center, Research & Development, Lexington, KY 40502

Correspondence should be addressed at * jgal2@uky.edu or [#] haining@uky.edu

[§] These authors contributed equally to this manuscript.

Supplemental Materials and Methods

RNA immunoprecipitation (RNA-IP) and quantitative PCR (Q-PCR)

293T cells were transfected with FLAG-tagged WT or F380L/F382L double mutant G3BP1 or FLAG vector. FLAG immunoprecipitations were performed as above with the inclusion of SUPERase-In RNase inhibitor (Ambion Life Technologies, AM2696) at 1:100 dilution. RNA was isolated from the total cellular extracts and FLAG eluates with TRIzol LS reagent (Life Technologies, 10296-010). The cDNAs were prepared using the SuperScript III First-Strand Synthesis System (Life Technologies, 18080-051) with random hexamer priming. Quantitative PCR was performed using Power SYBR Green PCR Master Mix (Life Technologies, 4367659) on an Agilent Mx3005P Q-PCR system. The Q-PCR primers were 5'-CACAAACTTGAACAGCTACGG-3' and 5'-GGTGATTGCTCAGGACATTTC-3' for c-myc, normalized to Rpl13a with primers 5'-GCCATCGTGGCTAAACAGGTA-3' and 5'-GTTGGTGTTCATCCGCTTGC-3'.

Supplemental Figures

Supplemental Figure 1. Co-localization of SOD1, TIA1 and G3BP1 in G93A transgenic mice spinal cord. Triple staining of SOD1, TIA1 and G3BP1 was performed using spinal cords of 90 days old G93A or WT SOD1 transgenic mice that were opposite gender from those in Figure 1A. G3BP1, TIA1 and mutant SOD1 were co-localized in inclusions while WT SOD1 shows even cytoplasmic distribution. Scale bars, 10μm.

Supplemental Figure 2. Primary antibody-omitted controls of the mouse spinal cord stainings in Figure 1A. No fluorescence signals were detected in samples with no primary antibodies, supporting the specificity of SOD1, TIA1 and G3BP1 antibodies used in this study. Scale bars, 10μm.

Supplemental Figure 3. Co-localization of SOD1, TIA1 and G3BP1 in fibroblast cells isolated from an L144F SOD1 ALS patient. Triple staining of SOD1, TIA1 and G3BP1 was performed using primary fibroblast cells derived from a familial ALS patient carrying L144F mutation. G3BP1, TIA1 and mutant SOD1 were co-localized in L144F mutant SOD1 inclusions while WT SOD1 shows even cytoplasmic distribution. Scale bars, 10μm.

Supplemental Figure 4. Confocal microscopic images of G3BP1, eIF3 and SOD1 in N2A cells expressing A4V mutant (top) or WT (bottom) SOD1. A4V mutant

SOD1, G3BP1 and eIF3 are co-localized in inclusions with the size of approximately 1-3 µm whereas WT SOD1 shows even cytoplasmic distribution. Scale bars, 10µm.

Supplemental Figure 5. Co-localization of A4V mutant SOD1 and G3BP1 in cytoplasmic inclusions in 293T cells. Cells were transfected with GFP-tagged A4V mutant (top) or WT (bottom) SOD1. SOD1 (green) and G3BP1 immunostaining (red) are visualized using a Leica SP5 confocal microscope. The top panel shows the co-localization of A4V mutant SOD1 and G3BP1 in inclusions (illustrated by arrows). The zoom-in inset shows a G3BP1 sheath around the mutant SOD1 inclusion. The bottom panel shows even cytoplasmic distribution of WT SOD1-EGFP. The nuclei were stained with DAPI. Scale bars, 10µm.

Supplemental Figure 6. F180 and F182 residues are critical to RNA binding of G3BP1. a. The consensus sequence of the RNP1 motif along with the corresponding RNP1 motif in human G3BP1. The conventional numbering of the positions in RNP1 [1] is shown in the top. Residues conserved in the respective positions of the RNP1 motif are shown in the top line and alternative conserved residues are shown in the second and third lines. The conserved positions in G3BP1 are highlighted in yellow. **b.** Quantification of RNA immunoprecipitated with WT or F380L/F382L G3BP1. RNA immunoprecipitation was performed using FLAG-tagged WT or F380L/F382L G3BP1 or vector control. Significantly reduced amount of endogenous c-myc mRNA was immunoprecipitated by F380L/F382L mutant G3BP1 as compared to WT G3BP1. The endogenous levels of c-myc mRNA were not significantly different between the extracts. The results of three independent parallel experiments are shown. * $p = 2.7 \times 10^{-4}$.

References

1 Maris C, Dominguez C, Allain FH (2005) The RNA recognition motif, a plastic RNA-binding platform to regulate post-transcriptional gene expression. FEBS J 272: 2118-2131 Doi 10.1111/j.1742-4658.2005.04653.x

Supplemental Figure 1

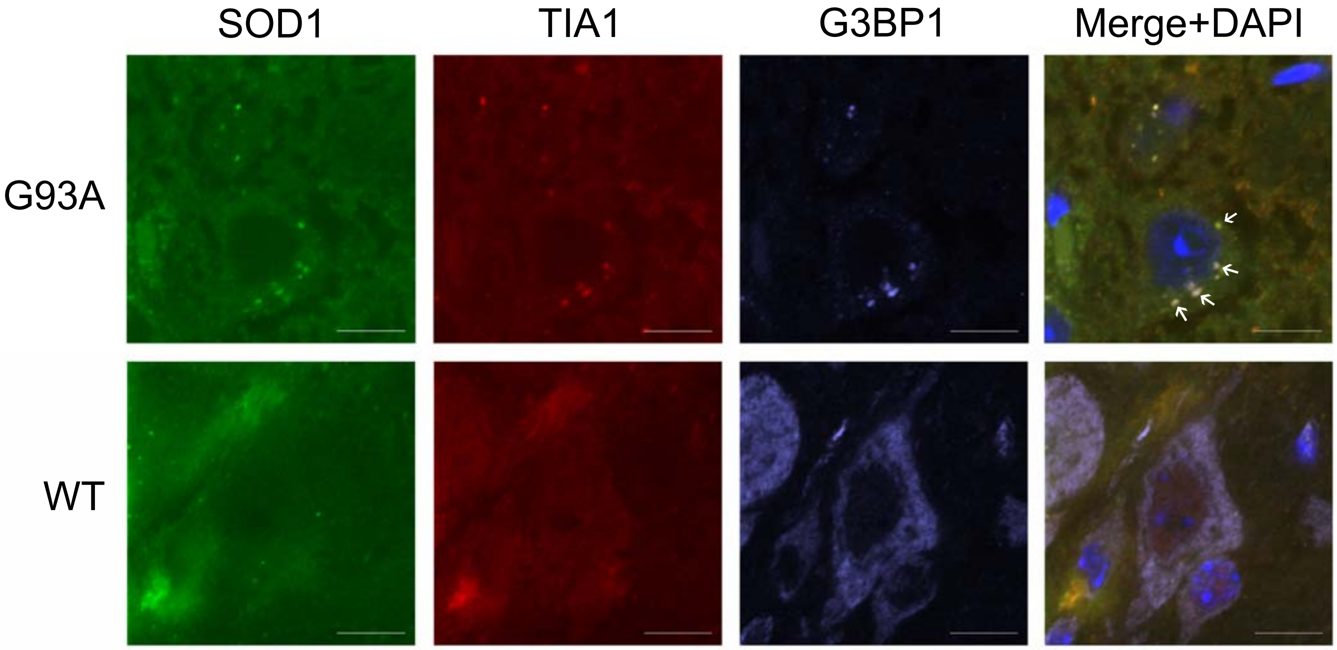

Supplemental Figure 2

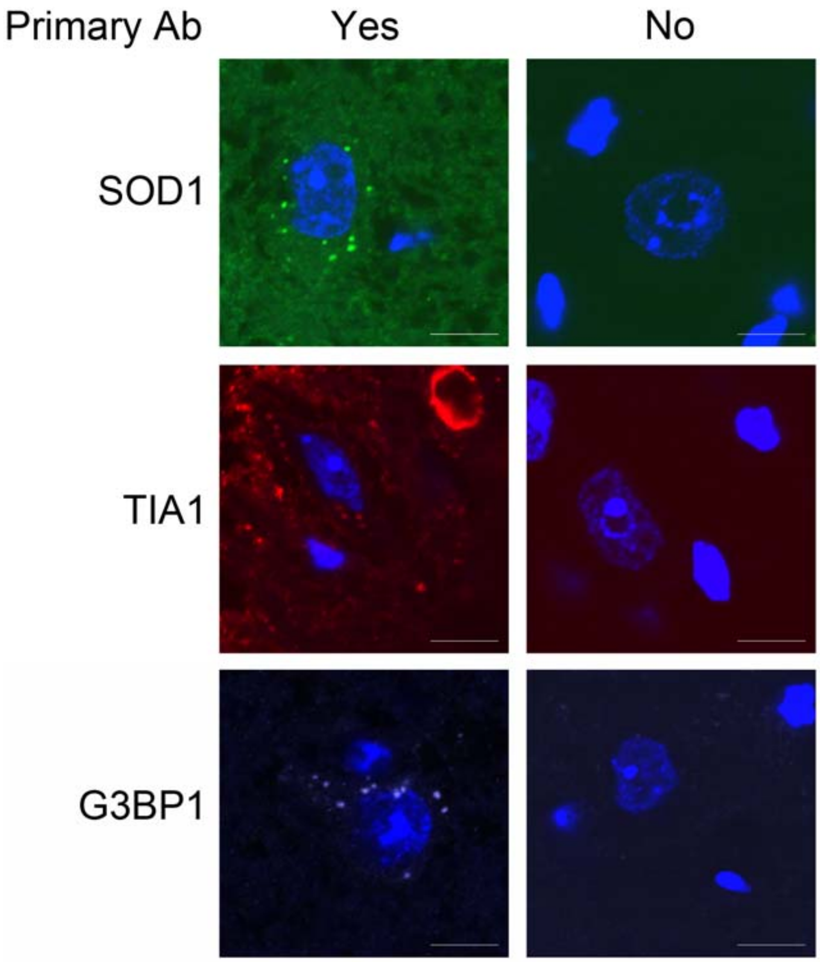

Supplemental Figure 3

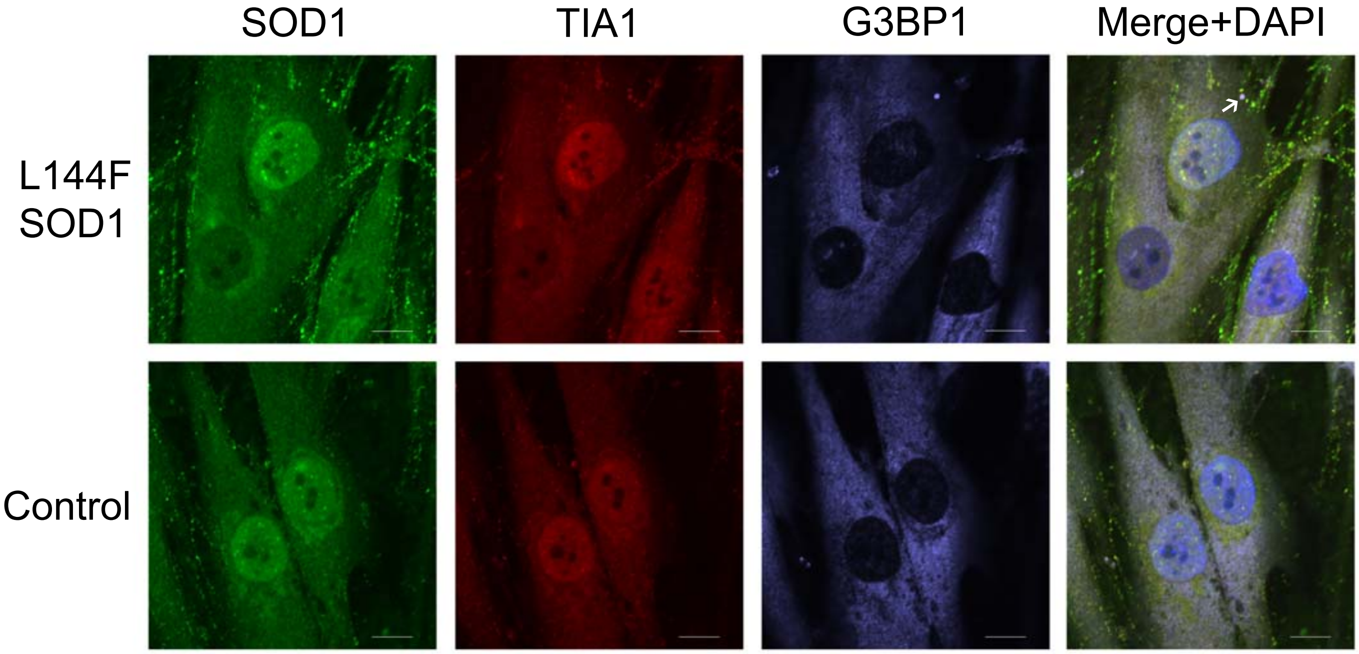

Supplemental Figure 4

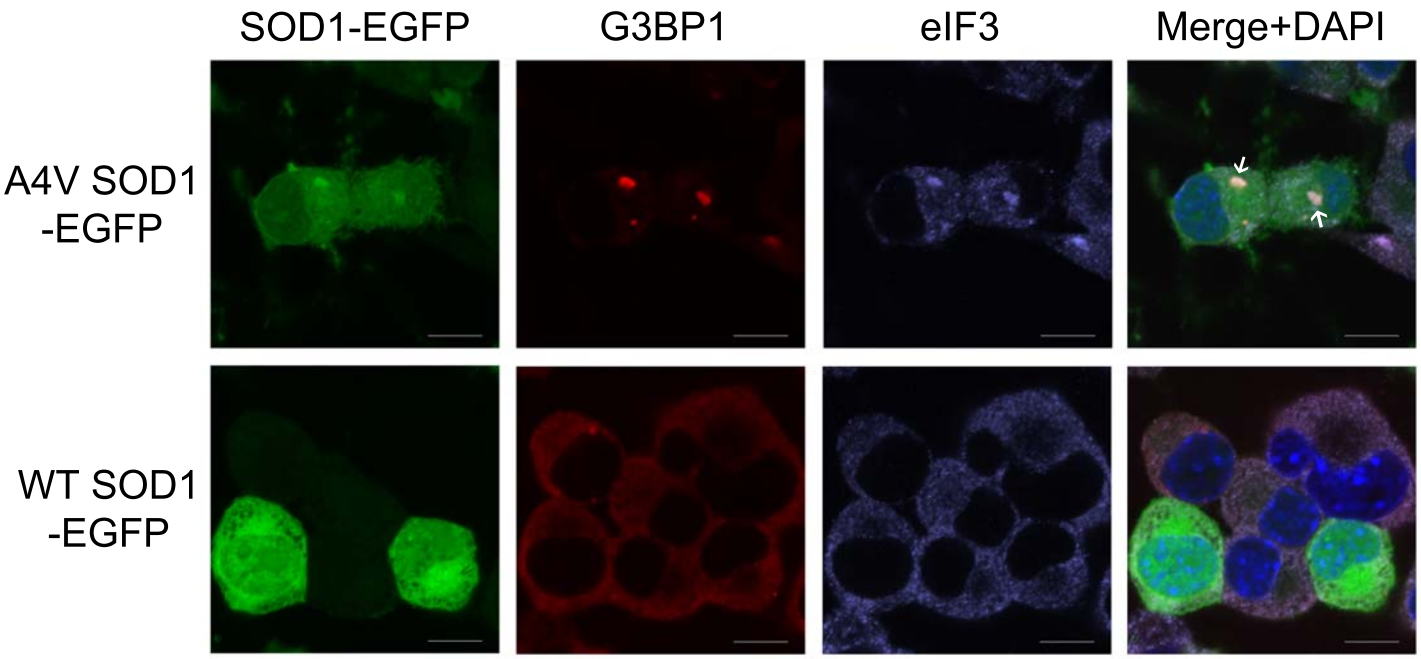

Supplemental Figure 5

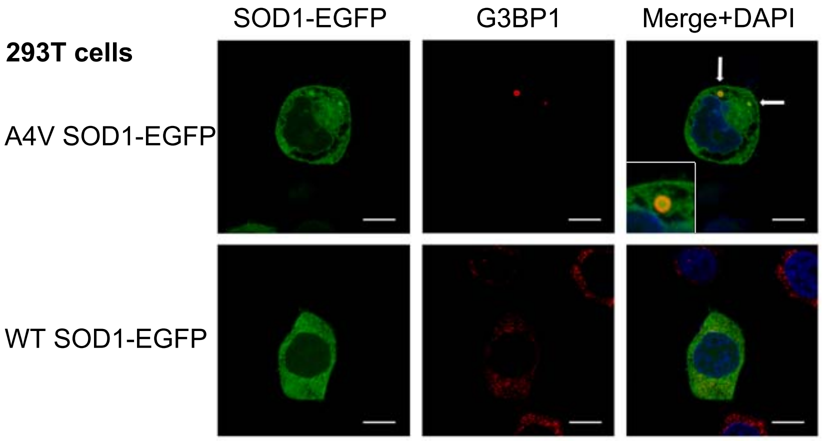

Supplemental Figure 6

a

```
                            12345678
                           [RGFGFIXF
RNP1 consensus             [K YAYL Y
                           [     V
G3BP1 RRM              378  PNFGFVVF 385
```

b

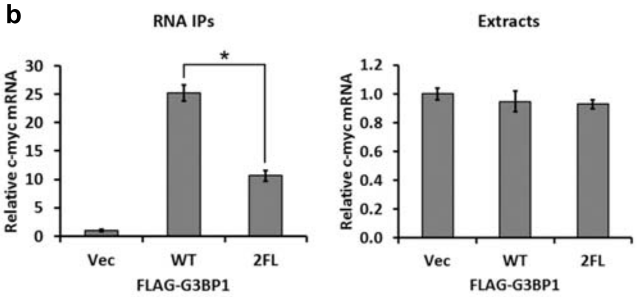

Supplement: Supplementary file 1 — Supplementary material 1 (PDF 366 kb) [file 401_2016_1601_MOESM1_ESM.pdf]
